# Mitochondrial serine protease Omi/HtrA2 accentuates brain ischemia/reperfusion injury in rats and oxidative stress injury in vitro by modulating mitochondrial stress proteins CHOP and ClpP and physically interacting with mitochondrial fusion protein OPA1

**DOI:** 10.1080/21655979.2020.1822105

**Published:** 2020-10-04

**Authors:** Hailong You, Yao Jin, Jinsong Kang, Ying Mao, Jing Su, Liankun Sun, Li Wang, Hao Meng

**Affiliations:** a Department of Pathogenobiology, Jilin University Mycology Research Center, College of Basic Medical Sciences, Jilin University, Changchun, China; b Department of Pathophysiology, College of Basic Medical Sciences, Jilin University, Changchun, China; c Department of Neurosurgery, The First Hospital of Jilin University, Changchun, China

**Keywords:** Brain ischemia, mitochondrial, omi/HtrA2, oxidative stress, reperfusion injury

## Abstract

Serine protease Omi/HtrA2, a member of the HtrA family, is closely related to the maintenance of mitochondrial integrity and participates in apoptosis but its role in cerebral ischemia/reperfusion (I/R) injury and cellular oxidative stress response remains unclear. In this study, we found that I/R injury resulted in a time-dependent increase in Omi/HtrA2 expression in rat brain tissue. Inhibition of Omi/HtrA2 significantly inhibited XIAP cleavage in H_2_O_2_-induced PC12 cells. In addition, inhibition of Omi/HtrA2 significantly inhibited the up-regulation of mitochondrial stress proteins CHOP and ClpP, significantly reduced mitochondrial aggregation, and attenuated the decline of mitochondrial ΔΨm in PC12 cells. Studies show that there is a physical interaction between Omi/HtrA2 and OPA1. We found that Omi/HtrA2 and OPA1 are closely related to the oxidative stress mitochondrial response in PC12 cells. The current study has demonstrated that Omi/HtrA2 is upregulated in brain I/R injury *in vivo* and is implicated in mitochondrial response to oxidative stress *in vitro* by regulating mitochondrial stress proteins CHOP and CLpP and by interacting with mitochondrial cristae remodeling protein OPA1. These findings suggest that Omi/HtrA2 could be a candidate molecular target in diseases that involve oxidative stress such as in I/R injury.

**Abbreviation**: ATP: Adenosine tripHospHate; Bax: BCL2-Associated X; Bcl-2: B-cell lympHoma-2; BSA: Albumin from bovine serum; DMEM: Dulbecco’s Minimum Essential Medium; DMSO: Dimethyl sulfoxide; HSP60: Heat shock protein60, 70; L-OPA1: Long forms of OPA1; Omi/HtrA2: high-temperature-regulated A2; MCAO: Middle cerebral artery occlusion; OPA1: Optic AtropHy; PBS: PHospHate buffered saline; PMSF: pHenylmethyl sulfonylfluoride; ROS: reactive oxygen species; SDS: Sodium dodecyl sulfate; S-OPA1: Short forms of OPA1; TTC: TripHenyltetrazalium chloride; XIAP: X-linked inhibitor apoptosis protein

## GRAPHICAL ABSTRACT

**Figure:**
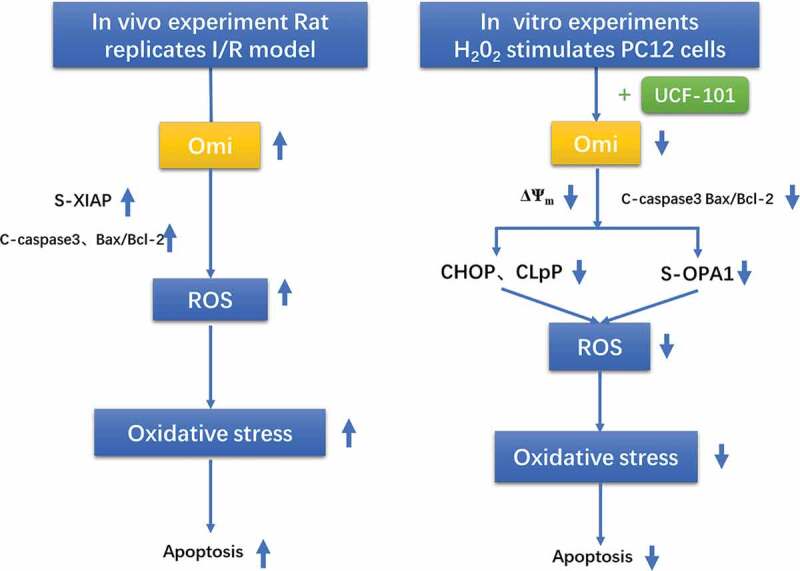

## Introduction

Brain ischemia is a common clinical condition and ischemia/reperfusion (I/R) injury may ensue following the restoration of blood supply to the brain tissues [1]. The mechanisms of brain I/R injury have not been fully elucidated and may partially involve neurocytotoxicities of excitatory amino acids, excessive production of free radicals, inflammation and overload of intracellular calcium. Brain I/R injury causes apoptosis and/or necrosis of neurons, depending on the duration and severity of ischemia. Longer duration of ischemia or severe ischemia causes necrosis of neurons while brief ischemia or mild ischemia induces apoptosis of neurons; furthermore, delayed neuron death following brain I/R injury mainly involves apoptosis of neurons [2].

Oxidative stress occurs when the generation of reactive oxygen species (ROS) and reactive nitrogen species (RNS) exceeds the capability of endogenous antioxidant systems [3,4]. Chemical stimuli like H_2_O_2_ causes oxidative stress and may activate the mitochondrial apoptotic pathway [5,6]. The mitochondria, which are the key determinants of cellular fate and capable of inducing cell death and are involved in oxidative stress-induced apoptosis, necrosis or necropolis [7,8] following brain I/R injury.

Omi/HtrA2, a member of the HtrA family, is intimately associated with maintaining mitochondria integrity. It is also involved in cellular death and, upon its release into the cytosol, participates in the caspase-dependent and independent apoptotic pathways [9–11]. Omi/HtrA2 induces apoptosis by directly binding to the inhibitor of apoptosis proteins (IAPs) and inhibiting IAP protease activity [12]. It degrades inhibitors of caspase-9, 3 and 7, allowing the release of active caspases [12]. When Omi/HtrA2 protease activities are blocked or inhibited, which prevents degradation of XIAP, apoptosis of ischemic myocardial cells becomes lessened due to inhibition of caspase activities by XIAP^22^. Omi/HtrA2 also participates in caspase-independent apoptosis [13,14].

Omi/HtrA2 protease is involved in maintaining the morphology and function of mitochondria in neurons [15] and loss of Omi/HtrA2 protease activity was found to cause neuromuscular disorder in *mnd2* mutant mice^37^. Omi/HtrA2 translocates from the mitochondria to the cytosol and participates in neuronal death in brain ischemia in rats [16]. Inhibition of Omi/HtrA2 by ucf-101 was found to reduce brain infarct volume in rats with middle cerebral artery occlusion (MCAO) and attenuate ischemia-induced increase in the amount of the cleavage products of active caspase-8 and caspase-3 [17]. Bax can destroy the integrity of mitochondrial membrane, release cytochrome C into the cytoplasm, and activate caspases 9, 3 and 7 through apoptotic protein activator to induce apoptosis.

Accumulation of misfolded or unfolded proteins in the matrix of mitochondria induces mitochondrial-unfolded protein reaction (UPR^mt^) [18–20] and leads to upregulation of heat shock proteins HSP60 and HSP10 and mitochondria protease ClpP [21]. OPA1 (optic atrophy 1) is a GTPase that regulates the dynamics of mitochondria and OPA1-dependent cristae remodeling stabilizes mitochondrial cristae, thus increasing mitochondrial respiratory efficiency, and blunts mitochondrial dysfunction, and attenuates ROS production [22,23]. OPA1 prevents proton leakage in energy metabolism and maintains the integrity of mitochondria membrane potential [24,25]. It also plays a crucial role in controlling cytochrome c release and mitochondrial fusion/fission during brain I/R [26]. Fusion of mitochondria involves both the long and short form of OPA1 (L- and S-OPA1). OPA1 plays a very important role in apoptosis in preventing cytochrome c release [27,28].

In the current study, we sought to determine the expression of Omi/HtrA2 in rat brain tissues following I/R injury using a rat MCAO model and further examined the role of Omi/HtrA2 in oxidative stress induced by H_2_O_2_ using PC12 cells.

## Materials and methods

### Cells and treatments

Rat adrenal medulla pheochromocytoma cell line PC12 (The Cell Bank of the Institute of Biochemistry and Cell Biology, Shanghai, China) was grown in high glucose DMEM (Gibco, Grand Island, NY, USA) containing fetal bovine serum (Sigma, St. Louis, MO, USA) and equine serum (Hyclone, South Logan, UT, USA) with appropriate antibiotics. Cells were treated with 250 µM H_2_O_2_ to induce apoptosis of PC12 cells in the presence or absence of 25 µM specific Omi/HtrA2 inhibitor ucf-101, or H_2_O_2_ plus ucf-101 as detailed elsewhere in the text.

### Animals

The study protocol was approved by the Ethics Committee of Jilin University and performed according to the National Institutes of Health Guidelines for the Care and Use of Laboratory Animals. Forty healthy male Wistar rats, weighing between 250 and 300 g each, were purchased from the Experimental Animal Center of Jilin University, Changchun, China. The rats were allowed two weeks to accommodate and had *ad libitum* access to laboratory chow.

### MCAO

The rat MCAO model was established as previously described with minor modifications [29,30]. The rats were anesthetized by intraperitoneal injection of 1% pentobarbital sodium (50 mg/kg). The middle cerebral artery was occluded for 1, 1.5 and 3 h, respectively, before the blood flow was restored, which was confirmed by laser Doppler. The sham-operated rats underwent the same surgical procedure except that the middle cerebral artery was not occluded. After the rats regained consciousness, they were evaluated using the Longa scale (Table 1) [30].

**Table 1.** Longa scale.

Level	Score	Content
I	0	Walking normally or without neurological deficits
II	1	The contralateral forepaw cannot be fully extended, or there is a slight neurological deficit
III	2	Turn contralateral or moderately neurologically defective
IV	3	Dumping to the opposite side, or severe neurological impairment
V	4	Inability to move spontaneously accompanied by low level of consciousness

### Longa scoring method neurological examination

To avoid confounding effects, we chose mice with Level 2 to Level 4 for the study (Table 2). In this state, the neurological score of mice was stable and the degree of interference was small.

**Table 2.** Longa scores of the study rats.

Group	Longa scores
Sham-operation	0.00 ± 0.00
Ischemia-1h	1.20 ± 0.422*
Ischemia-1.5h	2.00 ± 0.667**
Ischemia-3h	2.50 ± 0.527**

*P < 0.05, **P < 0.01 *vs*. Sham-operation.

### Evaluation of infarct area

The infarct volume was determined using 2% 2,3,5-triphenyltetrazolium chloride (TTC) staining. After 24 h of reperfusion, each rat was euthanized; the brain was removed, cut into 0.2-cm-thick sections, and stained at 37°C for 30 min with 2% TTC solution. A second section in the caudal side of each brain was also chosen to quantify the infarct area using NIH Imagine 1.6. The size of the infarct area was calculated by the percentage of the infarct area relative to the whole brain section area. Evaluation was undertaken by a technician who was blinded to group assignment of the study animals.

### Histological study

Rats with a Longa scale score of 3 were euthanized after anesthesia by intraperitoneal injection of 1% pentobarbital sodium (50 mg/kg) followed by transcardial perfusion with normal saline and fixation in 4% paraformaldehyde. The tissues were routinely dehydrated and transparentized. After paraffin-embedding, the tissues were cut into 4-μm-thick sections, followed by hematoxylin and eosin (H&E) staining. The sections were then observed under a light microscope and photographed.

### MTT assays and Trypan blue staining

Logarithmically growing PC12 cells were plated into 96-well plates (5 × 10^3^ cells/well) in quadruplicate for each group. Ten microliter MTT (at a final concentration of 5 mg/mL) was added into each well and after the cells were incubated for 4 h, the supernatant was discarded. DMSO (150 μL) was added to each well to dissolve the precipitate. After shaking for 2 min, absorbance was read at 490 nm using a microplate reader (BioRad, Hercules, CA, USA).

PC12 cells were rendered into single-cell suspensions after tryptic digestion (10^6^ cells/mL) and then routinely stained with 0.4% trypan blue. Live and dead cells were counted within 3 min and cell viabilities were calculated under an inverted microscope (Olympus, Tokyo, Japan).

### Western blotting assays

Cells were lysed using the RIPA lysis buffer containing 1% phenylmethanesulfonyl fluoride (PMSF) and β-mercaptoethanol. Protein concentration in the lysate was determined using the Bradford method. The proteins were resolved by PAGE electrophoresis. Immunoblotting assays were performed using a standard protocol. Primary antibodies against the following proteins were used: cleaved caspase 3 (0.5 μg/mL, MAB835, Bio-techne, Minneapolis, MN, USA), Bax (1:1000,sc-7480, Santa Cruz Biotechnology, Santa Cruz, CA, USA), Bcl-2 (1:1000, sc-7382, Santa Cruz Biotechnology), Omi/HtrA2 (1:500, sc-58371, Santa Cruz Biotechnology), XIAP (1:200, sc-55550, Santa Cruz Biotechnology), CLpP (1:500, 15698-1-AP, ProteinTech, Rocky Hill, NJ, USA), CHOP (1:500, 15204-1-AP, ProteinTech), OPA1 (1:500, sc-393296, Santa Cruz Biotechnology), and actin (1:1000, sc-58673, Santa Cruz Biotechnology). After incubation with secondary goat anti-rabbit (H + L) immunoglobulin (Ig) G (SA00001-2) and goat anti-mouse (H + L) IgG (SA00001-1) (ProteinTech), the protein bands were visualized by ECL (Pierce, Rockford, IL, USA). Densitometry was performed using the Kodak ID3.6 software.

### Measurement of mitochondrial ΔΨ_m_ using the JC-1 dye

Changes in ΔΨ_m_ were measured using a standard protocol [13]. Briefly, logarithmically growing PC12 cells were plated in a 6-well plate at a density of 3.5 × 10^4^ per well, incubated until the cells became 80%-85% confluent. The cells were then either left untreated (negative control) or treated with H_2_O_2_, or H_2_O_2_ plus ucf-101. Next, the cells were exposed to the fluorescent cationic dye JC-1 (5,5′,6,6′-tetrachloro-1,1′,3,3′-tetraethylbenzimidazolylcarbo-cyanine iodide) for 25 min before observation at green and red emission wavelengths using a fluorescence microscope (IX-71; Olympus Corporation, Tokyo, Japan).

After the addition of JC-1 and incubation in the dark, the cells were centrifuged again at 600 g for 5 min at 4°C. The pellet was suspended in 1 x buffer and washed and then suspended in appropriate media after centrifugation. The samples were analyzed using a BD Accuri C6 flow cytometer (BD Biosciences, Franklin Lakes, NJ, USA).

### Mitotracker staining

Logarithmically growing PC12 cells were plated in a 24-well plate at a density of 3.5 × 10^4^ per well and incubated until the cells became 80%-85% confluent. After appropriate drug treatment, the cells were stained with 50 nM Mitotracker for 20 min at room temperature (20°C). Green, blue and red fluorescence was observed under a confocal microscope at excitation wavelengths of 500, 340 and 579 nm, respectively, following the manufacturer’s instructions.

### Co-immunoprecipitations

Cells were lyzed in 200 μL NP40 lysis buffer containing 1% PMSF. The lysates were clarified by centrifugation and protein concentrations were determined using the BCA method (Beyotime Institute of Biotechnology, Haimen, China). Each sample was incubated with anti-HtrA2/Omi antibody (2 μg per 500 μg of total protein) (sc-58371, Santa Cruz Biotechnology) for 1 h at 4°C. Protein G Agarose Fast Flow (Beyotime Institute of Biotechnology) (20 μL) was added to each sample before overnight incubation at 4°C. The beads were collected by centrifugation and washed three times with PBS. The immune complexes were released from the beads by boiling with 5X SDS-PAGE loading buffer and analyzed by immunoblotting. The following antibodies were used in immunoblotting analyses: anti-OPA1 (1:500, sc-393296, Santa Cruz Biotechnology) antibody and goat anti-mouse (H + L) IgG conjugated secondary antibody (1:1000, SA00001-1, ProteinTech).

### Statistical analysis

Data were expressed as mean ± SD of at least three independent experiments and analyzed with SPSS19.0 (SPSS Inc., Chicago, IL, USA). Comparison between groups was done using one-way ANOVA with the two-sided Student’s *t* test. A *P* value of less than 0.05 was considered statistically signiﬁcant.

## Results

### Ischemia/reperfusion injury causes infarction and apoptosis of rat brain tissues

**Figure 1.**
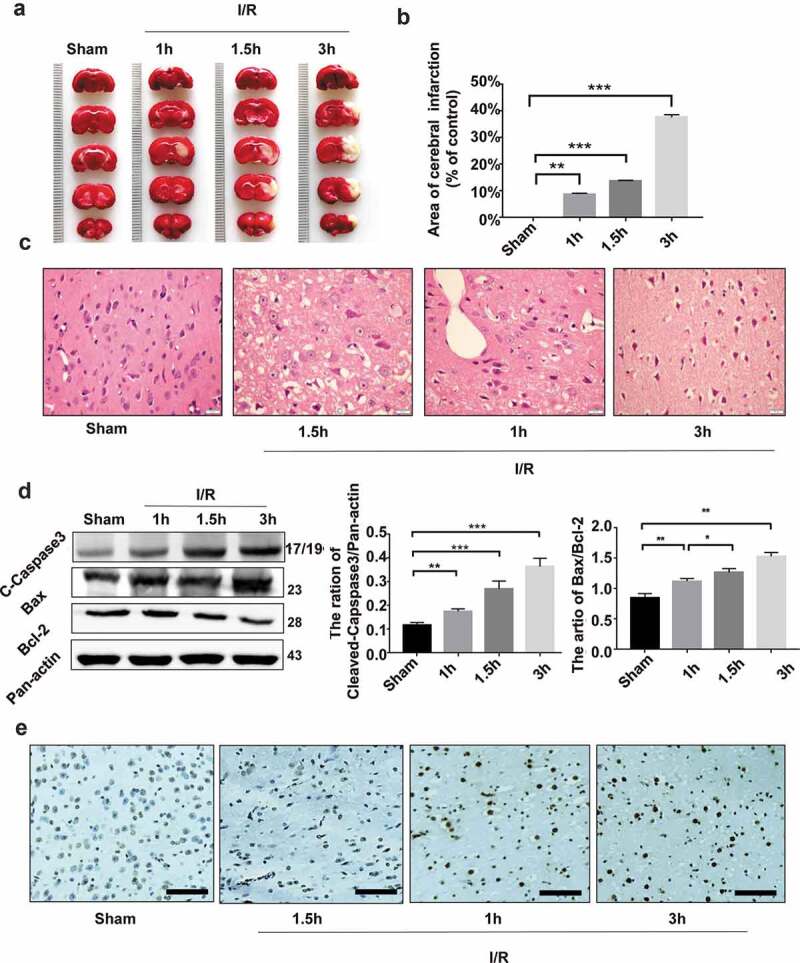
Ischemia/reperfusion injury causes infarction and apoptosis of rat brain tissues. The rat middle cerebral artery occlusion (MCAO) model was established as detailed in Methods. The MCA was occluded for 1, 1.5 and 3 h, respectively, followed by reperfusion for 24 h. (a) The photographs of the brain samples of rats undergoing sham operation or MCAO. (b) The infarct ratio of 2% TTC stain in each group. Data are presented as mean ± SD, n = 3. **P < 0.01 and ***P < 0.001 *vs*. the sham group. (c) H&E staining of the infarct marginal zone in the cerebral cortex. Bar = 20 μm. (d) Immunoblotting assays of cleaved caspase 3 and Bac/Bcl-2 in rat brain tissues undergoing ischemia/reperfusion injury. A representative immunoblot is shown (left) and Data are presented as mean ± SD, n = 3. *P < 0.05, **P < 0.01 and ***P < 0.001 *vs*. the sham group. (e) TUNEL staining of the infarct marginal zone in the brain cortex. Bar = 10 μm.

To examine whether brain I/R injury caused apoptosis of brain tissues, we established an MCAO model of brain I/R injury. The rats underwent sham operation or MCAO for 1, 1.5 and 3 h, respectively, followed by reperfusion for 24 h. TTC staining showed that the volume of the brain infarct area significantly increased over time following MCAO (*P* < 0.01 *vs*. the sham control group) (Figure 1(a,b)). H&E staining of brain sections revealed progressive changes typical of brain ischemia over time (Figure 1(c)). Consistently, immunoblotting assays demonstrated significant time-dependent increase in the levels of cleaved caspase 3 and Bac/Bcl-2 (*P* < 0.01 *vs*. the sham control group) (Figure 1(d)). TUNEL assays further showed significant time-dependent increase in the number of apoptotic cells in the brain infarct area (Figure 1(e)).

### Ischemia/reperfusion injury upregulates Omi/HtrA2 in brain tissues

**Figure 2.**
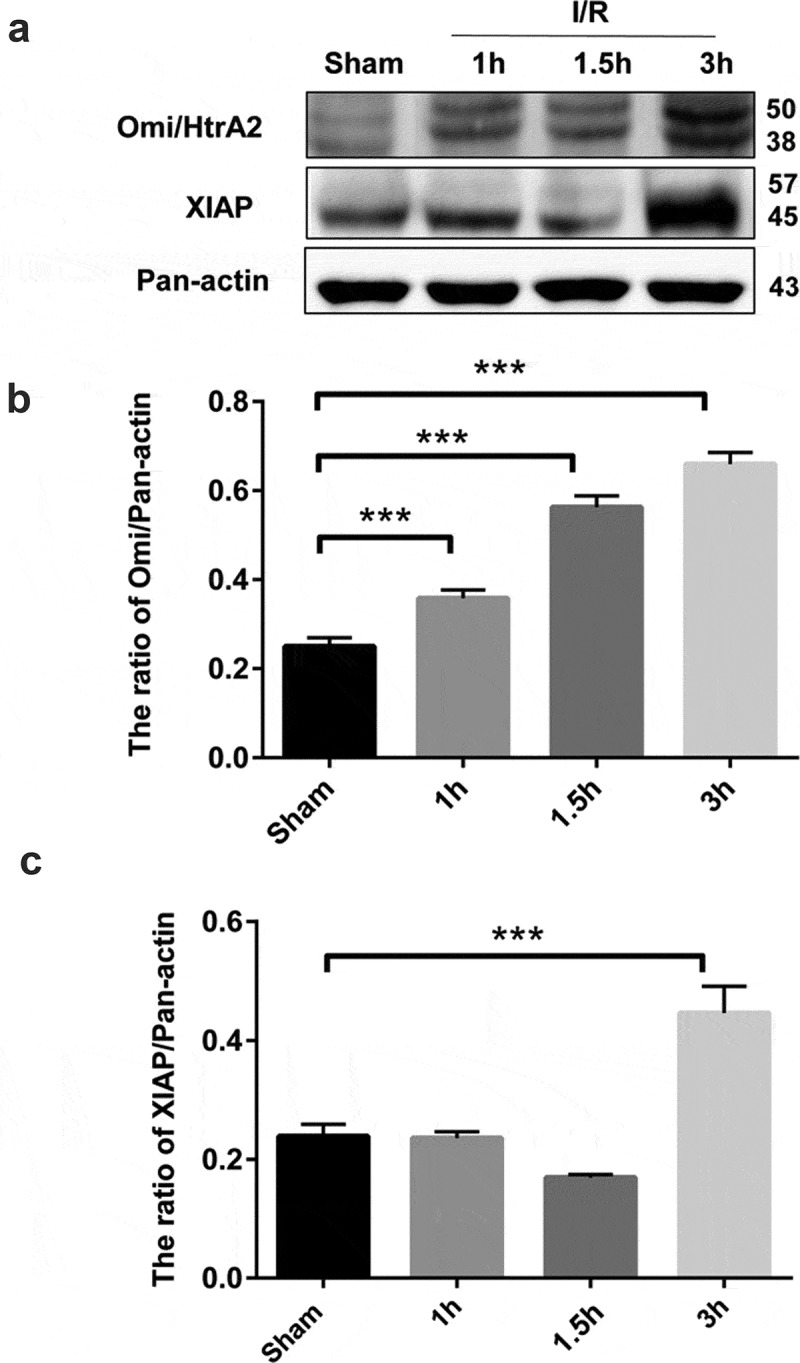
Ischemia/reperfusion injury upregulates Omi/HtrA2 in brain tissues. (a) Western blot analysis of Omi/HtrA2 and XIAP in brain tissues. A representative immunoblot is shown. The results of densitometric analysis are expressed as mean ± SD of three independent experiments and shown in bar graphs in (b) for Omi/HtrA2 and (c) for XIAP. Data are presented as mean ± SD, n = 3. ***P < 0.001 *vs*. the sham group.

Bax promotes the release of cytochrome c into the cytoplasm and activates caspases 9, 3 and 7 by activating apoptotic protein activator. Omi/HtrA2 is involved in caspase-dependent apoptosis and the proapoptotic mitochondrial serine protease translocates from the mitochondria to the cytosol after an apoptotic insult. We further examined the expression of Omi/HtrA2 in rat brain tissues undergoing I/R injury. Our immunoblotting assays demonstrated that I/R injury caused a significant time-dependent increase in the expression of Omi/HtrA2 in brain tissues (*P* < 0.01 *vs*. the sham control group) (Figure 2(a,b)). Furthermore, the expression of the apoptosis inhibitor XIAP was markedly higher at 3 h post I/R injury when compared to the control group (*P* < 0.01 *vs*. the sham control group) (Figure 2(a,c)).

### Omi/HtrA2 specific inhibitor ucf-101 attenuates H_2_O_2_-induced apoptosis of PC12 cells

**Figure 3.**
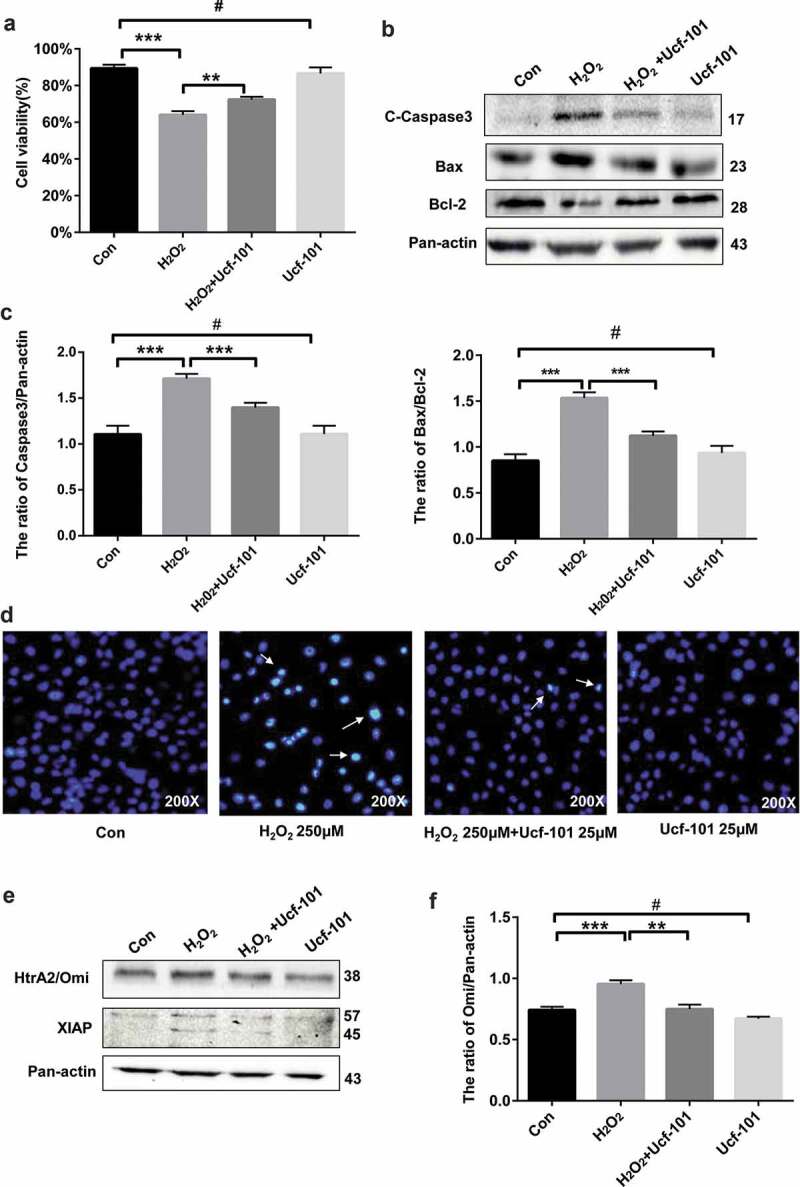
Omi/HtrA2 specific inhibitor ucf-101 attenuates H_2_O_2_ -induced apoptosis of PC12 cells. (a) Cell viability was determined by Trypan blue staining. Data are presented as mean ± SD (n = 3). ^#^*P* > 0.05 *vs*. the control group; ***P < 0.001 *vs*. the control group; **P < 0.01 *vs*. the H_2_O_2_ group. (b) Immunoblotting assays of cleaved caspase 3andBac/Bcl-2 in PC2 cells treated with H_2_O_2_ (250 μM), ucf-101 (25 μM) and H_2_O_2_ plus ucf-101 for 6 h. A representative immunoblot is shown. (c) Data are presented as mean ± SD, n = 3. ^#^*P* > 0.05 *vs*. the control group; ***P < 0.001 *vs*. the control group or the H_2_O_2_ group. (d) Hoechst staining of H_2_O_2_ treated cells. (e) Immunoblotting assays of Omi/HtrA2 and cleaved XIAP in PC2 cells treated with H_2_O_2_ (250 μM), ucf-101 (25 μM) and H_2_O_2_ plus ucf-101 for 6 h. A representative immunoblot is shown. (f) Data are presented as mean ± SD, n = 3. ^#^*P* > 0.05 *vs*. the control group; ***P < 0.001 *vs*. the control group; **P < 0.01 *vs*. the H_2_O_2_ group.

H_2_O_2_ is known to induce cytotoxicities of PC12 cells [31]. Our MTT assays showed that the IC_50_ of H_2_O_2_ was 250 μM (Figure S1) and Western blotting assays showed that H_2_O_2_ caused a significant dose-dependent increase in the rate of apoptotic PC12 cells (*P* < 0.01 *vs*. the control group). We treated PC12 cells with H_2_O_2_ (250 μM), ucf-101 (25 μM) and H_2_O_2_ plus ucf-101 for 6 h. Trypan blue staining showed that ucf-101 markedly abated the reduction in viabilities of PC12 cells by H_2_O_2_ (*P* < 0.01 *vs*. the H_2_O_2_ group) (Figure 3(a)). Western blot analysis further showed that ucf-101 significantly reduced H_2_O_2_-induced cleavage of caspase 3 and increase of Bax/Bcl-2 (*P* < 0.001 compared with the H_2_O_2_ group) (Figure 3(b,c)). Hoechst staining revealed that H_2_O_2_ noticeably increased the number of apoptotic cells, which was markedly attenuated by treatment with ucf-101 (D). We further examined the effect of Omi/HtrA2 specific inhibitor ucf-101 on Omi/HtrA2 expression in PC12 cells under oxidative stress by H_2_O_2_. Western blotting assays showed that ucf-101 markedly attenuated H_2_O_2_-induced upregulation of Omi/HtrA2 in PC12 cells (*P* < 0.01) (Figure 3(e,f)). In addition, ucf-101 apparently abated H_2_O_2_-induced cleavage of XIAP in PC12 cells (Figure 3(e)).

### Omi/HtrA2 inhibition alleviates H_2_O_2_-induced mitochondrial stress in PC12 cells

**Figure 4.**
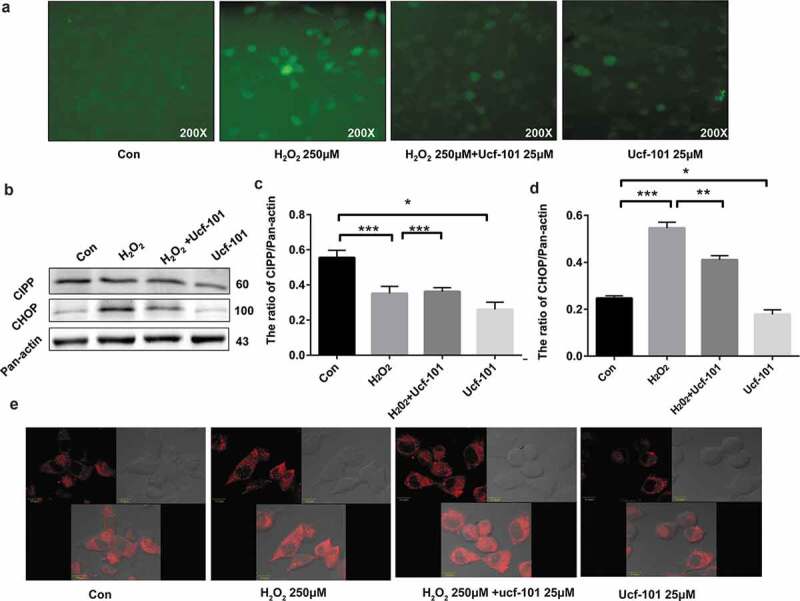
Omi/HtrA2 inhibition alleviates H_2_O_2_-induced clustering of mitochondria in PC12 cells. The cells were treated with H_2_O_2_ (250 μM), ucf-101 (25 μM) and H_2_O_2_ plus ucf-101 for 6 h and then stained with Mito-tracker Red. (a) ROS production of cells treated with H_2_O_2_ (250 μM), ucf-101 (25 μM) and H_2_O_2_ plus ucf-101 for 6 h. (b) S0033 Immunoblotting assays of CHOP and ClpP in PC2 cells treated with H_2_O_2_ (250 μM), ucf-101 (25 μM) and H_2_O_2_ plus ucf-101 for 6 h. A representative immunoblot is shown. (c, d). The results of densitometric analysis are expressed as mean ± SD, n = 3. ^#^*P* > 0.05 *vs*. the control group; ***P < 0.001 *vs*. the control group; **P < 0.01 *vs*. the H_2_O_2_ group. (e) Mitochondrial morphology is observed by laser scanning confocal microscopy (10.0 μm).

We further investigated the effect of Omi/HtrA2 by ucf-101 on stress response proteins in the mitochondria of PC12 cells under oxidative stress by H_2_O_2_. Examination of ROS production showed that H_2_O_2_ noticeably increased ROS production which was attenuated by treatment with ucf-101 (Figure 4(a)). Western blotting assays revealed that, compared to the control group, H_2_O_2_ caused a significant increase in the levels of CHOP and ClpP in PC12 cells, indicating that H_2_O_2_ caused significant mitochondrial stress (*P* < 0.001 *vs*. the control group) (Figure 4(b–d)). However, ucf-101 significantly attenuated H_2_O_2_-induced upregulation of CHOP and ClpP in PC12 cells (*P* < 0.01 or 0.001). We also tracked changes of mitochondria in PC12 cells using Mito-tracker Red. Our confocal microscopy revealed that, compared to the control group, H_2_O_2_ noticeably increased the clustering of mitochondria in PC12 cells (Figure 4(e)), indicating disruption of the mitochondria by H_2_O_2_. Omi/HtrA2 specific inhibitor ucf-101 apparently reduced mitochondria clustering.

### *Omi/HtrA2 inhibition partially attenuates H_2_O_2_-induced decline in* ΔΨ_m_ *in PC12 cells*

**Figure 5.**
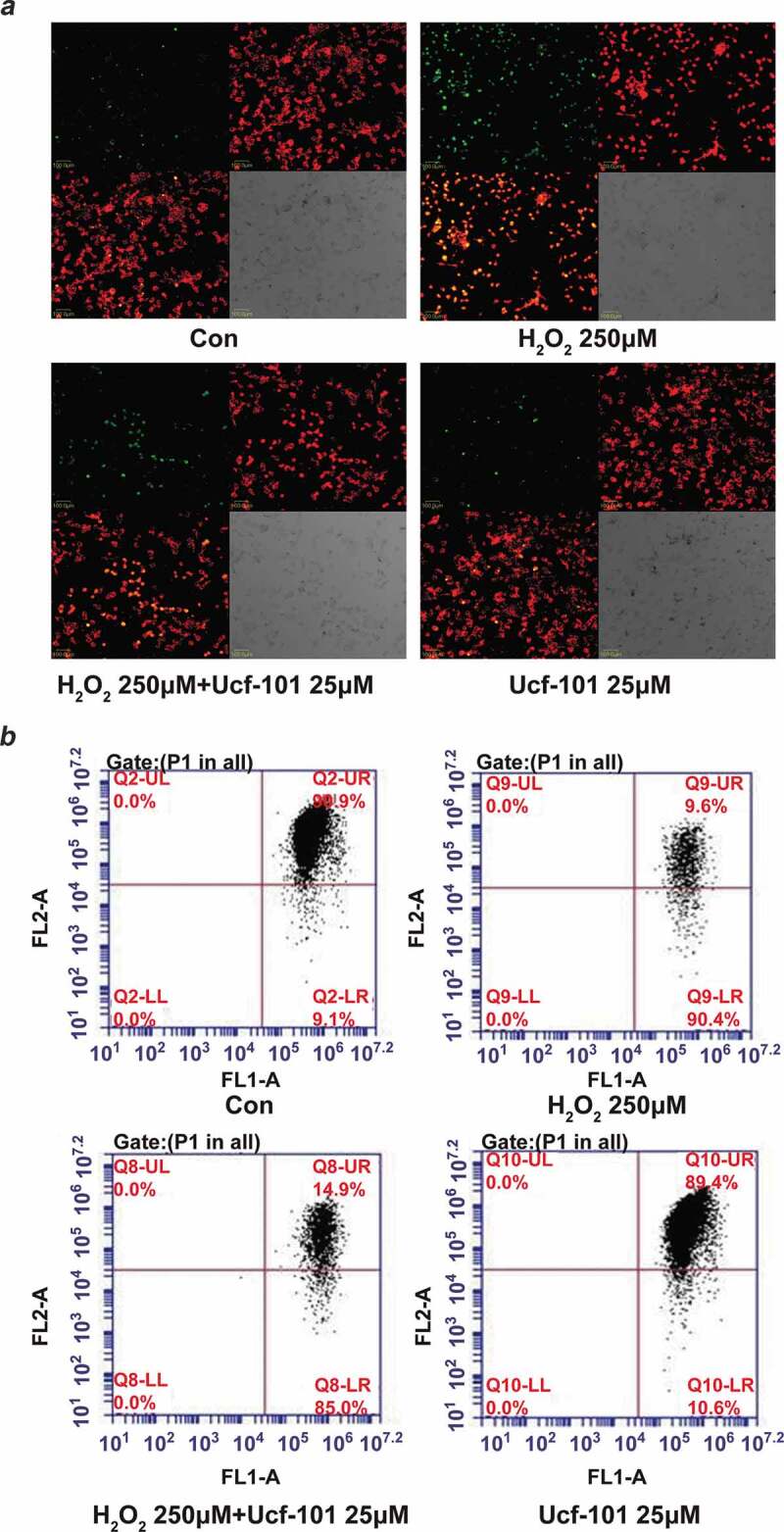
Omi/HtrA2 inhibition partially attenuates H_2_O_2_-induced decline in ΔΨ_m_ in PC12 cells. PC12 cells were treated with H_2_O_2_ (250 μM), ucf-101 (25 μM) and H_2_O_2_ plus ucf-101 for 6 h and then stained with JC-1 dye. (a) Changes in mitochondrial ΔΨ_m_ in PC12 cells were observed using by laser scanning confocal microscopy. 100.0 μm. (b) Changes in mitochondrial ΔΨ_m_ were further evaluated by flow cytometry. The light of FL1-A is green while the FL2-A is red.

We then sought to investigate the effect of Omi/HtrA2 on changes in mitochondrial ΔΨ_m_ using the JC-1 dye in PC12 cells treated with H_2_O_2_. We found that H_2_O_2_ treatment caused a noticeable reduction in mitochondrial ΔΨ_m_ as reflected by the apparent increase in blue fluorescence intensity (Figure 5(a)). By contrast, Omi/HtrA2 inhibition by ucf-101 abated H_2_O_2_-induced reduction in mitochondrial ΔΨ_m_ in PC12 cells.

Furthermore, flow cytometric analysis showed that H_2_O_2_ caused a significant decline in mitochondrial ΔΨ_m_ (9.6% *vs*. control 90.9%, *P* < 0.001 (Figure 5(b)). Co-treatment with ucf-101 partially alleviated the decline in mitochondrial ΔΨ_m_ (14.9% *vs*. H_2_O_2_ 9.6%., *P* < 0.001).

### Omi/HtrA2 inhibition partially disrupts H_2_O_2_-induced physical interaction between Omi/HtrA2 and OPA1

**Figure 6.**
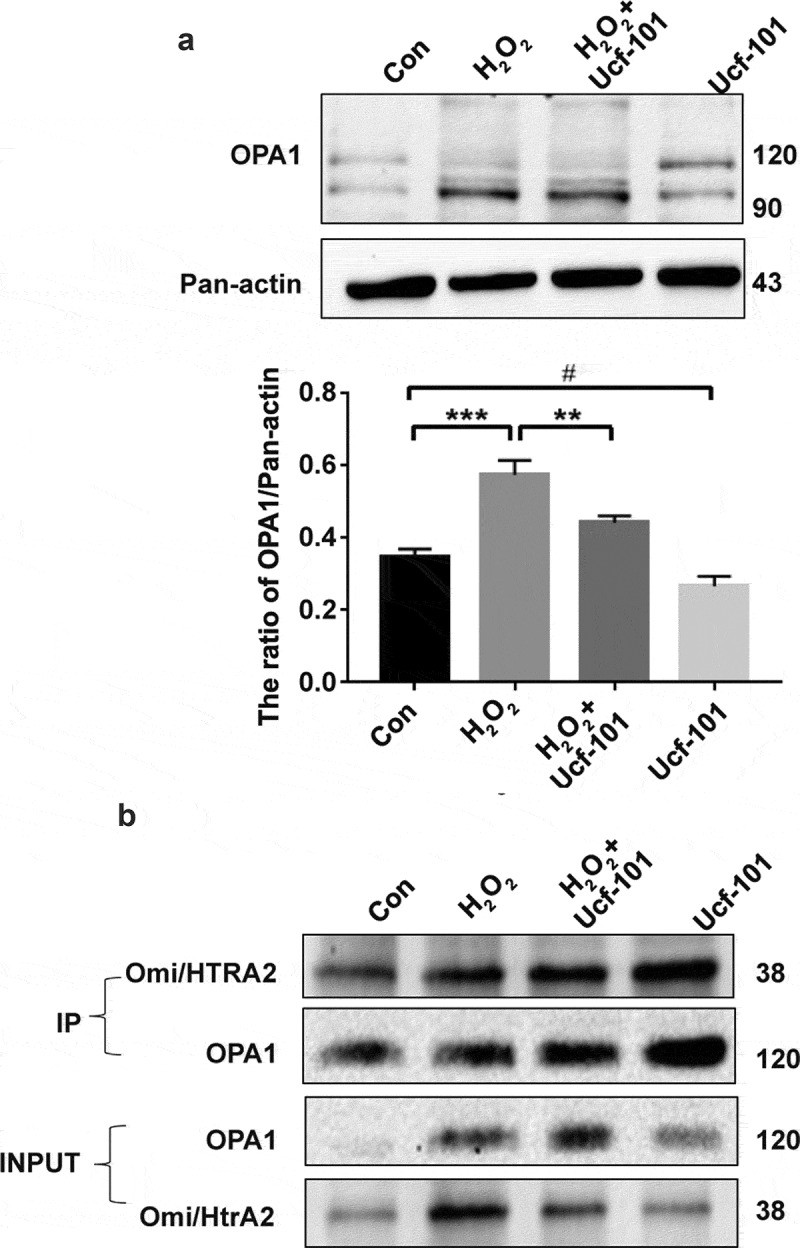
Omi/HtrA2 inhibition partially disrupts Omi/HtrA2 and OPA1 interaction in PC2 cells treated with H_2_O_2_. (a) Immunoblotting assays of the long (L-OPA1) and short OPA1 (S-OPA1) in PC2 cells treated with H_2_O_2_ (250 μM), ucf-101 (25 μM) and H_2_O_2_ plus ucf-101 for 6 h. A representative immunoblot is shown. Data are presented as mean ± SD (n = 3). ^#^*P* > 0.05 *vs*. the control group; ***P < 0.001 *vs*. the control group; **P < 0.01 *vs*. the H_2_O_2_ group. (b) Co-immunoprecipitation of endogenous Omi/HtrA2 and OPA1 in lysates of PC2 cells treated with H_2_O_2_ (250 μM), ucf-101 (25 μM) and H_2_O_2_ plus ucf-101 for 6 h. Lysates were subjected to Western blot analysis directly (inputs) or after incubation overnight with either protein G agarose alone (−) or with anti-OPA1 bound to protein G agarose (+). Immunoblots were probed with anti-OPA1 and anti-Omi/HtrA2 as indicated.

OPA1 controls the cristae remodeling arm of mitochondrial apoptosis [32]. Kieper et al. found that Omi/HtrA2 interacted with OPA1 in mitochondrial function and morphology [33]. Our Western blotting assays showed that compared to the control group, in H_2_O_2_ treated cells, there was a marked reduction in *L*-OPA1 expression (*P < *0.001) while there was a significant increase in *S*-OPA1 expression (*P* < 0.01) (Figure 6(a,b)). Noticeably, Omi/HtrA2 inhibition by ucf-101 significantly attenuated H_2_O_2_-induced increase in the expression of *S*-OPA1 (*P* < 0.01). Kieper et al. found that Omi/HtrA2 interacted with OPA1 in mitochondrial function and morphology [33]. Our co-immunoprecipitation assays showed that H_2_O_2_ significantly increased the physical interaction between Omi/HtrA2 and OPA1, which, however, was partially disrupted by treatment with ucf-101 (Figure 6(c)).

## Discussion

In the current study, we demonstrated that Omi/HtrA2 expression was significantly elevated in brain tissues of rats with I/R injury. We further showed that Omi/HtrA2 promoted cellular apoptosis in cells undergoing oxidative stress *in vitro* by interacting with OPA1 and participating in mitochondrial stress. Our findings revealed a critical role of Omi/HtrA2 in controlling cellular response to mitochondria stress in neurons undergoing I/R injury and in cells subject to oxidative stress.

Energy metabolic disturbance and increase production of ROS are important mechanisms of injury in ischemic stroke. H_2_O_2_ may cause an increase in intracellular ROS, thereby inducing oxidative stress injury. Mitochondrial membrane potential is disrupted, resulting in increased membrane permeability and leading to release of proapoptotic factors from the mitochondria into the cytosol and causing neuron injury *via* caspase-dependent and independent pathways. Omi/HtrA2 plays a dual role in the mitochondria by participating in apoptosis and maintaining mitochondria morphology and function. Normally Omi/HtrA2 in the mitochondria cristae promotes mitochondrion viability by maintaining mitochondrial morphology. The permeability of the outer membrane of the mitochondria decreases in response to outside stimuli, and Omi/HtrA2 is released into the cytosol and promotes apoptosis *via* the caspase-dependent and independent pathways. Omi/HtrA2 as a serine protease interacts with numerous proteins including anti-apoptotic protein XIAP [34,35]. The current study found that XIAP levels also increased along with upregulation of Omi/HtrA2 post I/R injury. XIAP is a substrate of Omi/HtrA2 and undergo cleavage by Omi/HtrA2 and loses its apoptosis inhibitory activities, thus serving as an indicator of Omi/HtrA2 activation. Our finding suggests that Omi/HtrA2 is upregulated and undergoes activation in response to I/R injury.

Recent studies have shown that mitochondria stress response occurs far in advance of autophagy and apoptosis [36,37]. CHOP is a marker of mitochondrial stress while ClpP generates a protein fragment that serves as a danger signal during mitochondrial stress [38]. Our Mito-tracker Red assays showed apparent clustering of mitochondria in PC12 cells in response to oxidative stress by H_2_O_2_. Our immunoblotting assays also revealed significant upregulation of CHOP and ClpP in PC12 cells, indicating the presence of marked mitochondrial stress. This was further proven by apparent reduction in mitochondrial ΔΨ_m_ in PC12 cells in response to oxidative stress by H_2_O_2_. This, however, was markedly attenuated by Omi/HtrA2 inhibition by ucf-101, suggesting that Omi/HtrA2 also participated in early mitochondrial stress response.

OPA1 is a mitochondrial inner membrane fusion protein and implicated in mitochondrial cristae remodeling. It is also intimately involved in apoptosis. Our immunoblotting assays revealed that Omi/HtrA2 was upregulated in response to oxidative stress by H_2_O_2_. Meanwhile, OPA1 underwent cleavage from L-OPA1to S-OPA1, which was attenuated by inhibition of Omi/HtrA2 by ucf-101. The co-immunoprecipitations further demonstrated increased physical interaction between Omi/HtrA2 and OPA1. These findings suggested that Omi/HtrA2 and OPA1 were intimately involved in mitochondrial response to oxidative stress in PC12 cells. The mechanisms whereby Omi/HtrA2 and OPA1 interact with each other in regulating mitochondrial stress response and apoptotic response remain to be further elucidated.

## Conclusion

In conclusion, the current study has demonstrated that Omi/HtrA2 is upregulated in brain I/R injury *in vivo* and implicated in mitochondrial response to oxidative stress *in vitro* by regulating mitochondrial stress proteins CHOP and ClpP and by interacting with mitochondrial cristae remodeling protein OPA1. These findings suggest that Omi/HtrA2 could be a candidate molecular target in diseases that involve oxidative stress.

## Supplementary Material

Supplemental Material

## Data Availability

The datasets used and/or analyzed during the current study are available.

## Highlights

- Omi/HtrA2 is involved in ischemia and reperfusion-induced brain injury.
- Omi/HtrA2 reduces mitochondrial membrane potential and regulates CHOP and CLPP.
- Omi/HtrA2 enhances ROS and aggravates oxidative stress damage.
- Omi/HtrA2 interacts with OPA1 and maintains mitochondrial homeostasis.

## References

[1] Lo EH, Dalkara T, Moskowitz MA. Mechanisms, challenges and opportunities in stroke. Nat Rev Neurosci. 2003;4(5):399–415. 12728267 10.1038/nrn1106

[2] Solaroglu I, Tsubokawa T, Cahill J, et al. Anti-apoptotic effect of granulocyte-colony stimulating factor after focal cerebral ischemia in the rat. Neuroscience. 2006;143(4):965–974. 17084035 10.1016/j.neuroscience.2006.09.014 PMC1820637

[3] Durackova Z. Some current insights into oxidative stress. Physiol Res. 2010;59(4):459–469. 19929132 10.33549/physiolres.931844

[4] Miyamoto H, Doita M, Nishida K. Effects of cyclic mechanical stress on the production of inflammatory agents by nucleus pulposus and annulus fibrosus derived cells in vitro. Spine. 2006;31(1):4. 16395168 10.1097/01.brs.0000192682.87267.2a

[5] Goping IS, Gross A, Lavoie JN, et al. Regulated targeting of BAX to mitochondria. J Cell Biol. 1998;143(1):207–215. 9763432 10.1083/jcb.143.1.207 PMC2132805

[6] Kelekar A, Thompson CB. Bcl-2-family proteins: the role of the BH3 domain in apoptosis. Trends Cell Biol. 1998;8(8):324–330. 9704409 10.1016/s0962-8924(98)01321-x

[7] Mei Y, Yongjiu L, Xiaocui T, et al. Neuroprotective effect of β-caryophyllene on cerebral ischemia-reperfusion injury via regulation of necroptotic neuronal death and inflammation: in vivo and in vitro. Front Neurosci. 2017;11:583. 29123466 10.3389/fnins.2017.00583 PMC5662640

[8] Vieira M, Fernandes J, Carreto L, et al. Ischemic insults induce necroptotic cell death in hippocampal neurons through the up-regulation of endogenous RIP3. Neurobiol Dis. 2014;68:26–36. 24746856 10.1016/j.nbd.2014.04.002

[9] Hegde R, Srinivasula SM, Zhang Z, et al. Identification of Omi/HtrA2 as a mitochondrial apoptotic serine protease that disrupts inhibitor of apoptosis protein-caspase interaction. J Biol Chem. 2002;277(1):432–438. 10.1074/jbc.M109721200 11606597

[10] Kuninaka S, Iida S-I, Hara T, et al. Serine protease Omi/HtrA2 targets WARTS kinase to control cell proliferation. Oncogene. 2007;12;26(17):2395–2406. 10.1038/sj.onc.1210042 17130845

[11] Vande Walle L, Lamkanfi M, Vandenabeele P. The mitochondrial serine protease HtrA2/Omi: an overview. Cell Death Differ. 2008;15(3):453–460. 18174901 10.1038/sj.cdd.4402291

[12] Srinivasula SM, Gupta S, Datta P, et al. Inhibitor of apoptosis proteins are substrates for the mitochondrial serine protease Omi/HtrA2. J Biol Chem. 2003;278(34):31469–31472. 12835328 10.1074/jbc.C300240200

[13] Cilenti L, Soundarapandian MM, Kyriazis GA, et al. Regulation of HAX-1 anti-apoptotic protein by Omi/HtrA2 protease during cell death. J Biol Chem. 2004;279(48):50295–50301. 15371414 10.1074/jbc.M406006200

[14] Liu Z, Li H, Derouet M, et al. Oncogenic ras inhibits anoikis of intestinal epithelial cells by preventing the release of a mitochondrial pro-apoptotic protein Omi/HtrA2 into the cytoplasm. J Biol Chem. 2006;281(21):14738–14747. 16461771 10.1074/jbc.M508664200

[15] Dagda RK, Chu CT. Mitochondrial quality control: insights on how Parkinson’s disease related genes PINK1, parkin, and Omi/HtrA2 interact to maintain mitochondrial homeostasis. J Bioenerg Biomembr. 2009;41(6):473–479. 20012177 10.1007/s10863-009-9255-1 PMC2809778

[16] Althaus J, Siegelin MD, Dehghani F, et al. The serine protease Omi/HtrA2 is involved in XIAP cleavage and in neuronal cell death following focal cerebral ischemia/reperfusion. Neurochem Int. 2007;50(1):172–180. 16978742 10.1016/j.neuint.2006.07.018

[17] Su D, Su Z, Wang J, et al. UCF‐101, a novel Omi/HtrA2 inhibitor, protects against cerebral ischemia/reperfusion injury in rats. Anat Rec (Hoboken). 2009;292(6):854–861. 19462455 10.1002/ar.20910

[18] Pagliarini DJ, Calvo SE, Chang B, et al. A mitochondrial protein compendium elucidates complex I disease biology. Cell. 2008;134(1):0–123. 10.1016/j.cell.2008.06.016 PMC2778844 18614015

[19] Papa L, Germain D. Estrogen receptor mediates a distinct mitochondrial unfolded protein response. J Cell Sci. 2011;124(9):1396–1402. 21486948 10.1242/jcs.078220 PMC3078808

[20] Pellegrino MW, Nargund AM. Signaling the mitochondrial unfolded protein response. Biochim Biophys Acta. 2012;1833(2):410–416. 22445420 10.1016/j.bbamcr.2012.02.019 PMC3393825

[21] Baker MJ, Tatsuta T, Langer T. Quality control of mitochondrial proteostasis. Cold Spring Harb Perspect Biol. 2011;3(7) 10.1101/cshperspect.a007559 PMC3119916 21628427

[22] Nie W, Zhang G, Hu Y. Research Progress of OPA1. Progress Mod Biomed. 2014;12:2394–2396.

[23] Varanita T, Soriano ME, Romanello V, et al. The Opa1-dependent mitochondrial cristae remodeling pathway controls atrophic, apoptotic, and ischemic tissue damage. Cell Metab. 2015;21(6):834–844. 26039448 10.1016/j.cmet.2015.05.007 PMC4457892

[24] Chen H, Chan DC. Emerging functions of mammalian mitochondrial fusion and fission. Hum Mol Genet. 2005;14(suppl_2):R283–R289. 16244327 10.1093/hmg/ddi270

[25] Twig G, Hyde B, Shirihai OS. Mitochondrial fusion, fission and autophagy as a quality control axis: the bioenergetic view. Biochim Biophys Acta. 2008;1777(9):1092–1097. 18519024 10.1016/j.bbabio.2008.05.001 PMC3809017

[26] Kumar R, Bukowski MJ, Wider JM, et al. Mitochondrial dynamics following global cerebral ischemia. Mol Cell Neurosci. 2016;76(Complete):68–75. 27567688 10.1016/j.mcn.2016.08.010 PMC5056829

[27] Van Blerkom J. Mitochondria in early mammalian development. Semin Cell Dev Biol. 2009;20(3):0–364. 10.1016/j.semcdb.2008.12.005 19136067

[28] Winkler-Stuck K, Kirches E, Mawrin C, et al. Re-evaluation of the dysfunction of mitochondrial respiratory chain in skeletal muscle of patients with Parkinson’s disease. J Neural Transm (Vienna). 2005;112(4):499–518. 15340872 10.1007/s00702-004-0195-y

[29] Garcia J, Wagner S, Liu K, et al. Neurological deficit and extent of neuronal necrosis attributable to middle cerebral artery occlusion in rats. Stroke. 1995;26(4):627. 7709410 10.1161/01.str.26.4.627

[30] Longa EZ, Weinstein PR, Carlson S, et al. Reversible middle cerebral artery occlusion without craniectomy in rats. Stroke. 1989;20(1):84–91. 2643202 10.1161/01.str.20.1.84

[31] Yang X, Mao X, Ding X, et al. miR-146a down-regulation alleviates H2O2-induced cytotoxicity of PC12 cells by regulating MCL1/JAK/STAT pathway. Cell Biol Toxicol. 2018;34(6):479–489. 29484526 10.1007/s10565-018-9424-2

[32] Frezza C, Cipolat S, Brito O. OPA1 controls apoptotic cristae remodeling independently from mitochondrial fusion. Cell. 2006;126(1):0–189. 10.1016/j.cell.2006.06.025 16839885

[33] Kieper N, Holmström KM, Ciceri D, et al. Modulation of mitochondrial function and morphology by interaction of Omi/HtrA2 with the mitochondrial fusion factor OPA1. Exp Cell Res. 2010;316(7):1213–1224. 20064504 10.1016/j.yexcr.2010.01.005 PMC3063334

[34] Guan F, Zhou L, Zhang C, et al. Effect of CCCP on endothelial cells mitochondrial membrane potential by JC-1 single staining detection. J Jilin Med Coll. 2014;35(5):324–326.

[35] Suzuki Y, Takahashi-Niki K, Akagi T, et al. Mitochondrial protease Omi/HtrA2 enhances caspase activation through multiple pathways. Cell Death Differ. 2004;11(2):208–216. 14605674 10.1038/sj.cdd.4401343

[36] Haynes CM, Ron D. The mitochondrial UPR - protecting organelle protein homeostasis. J Cell Sci. 2010;123(22):3849–3855. 21048161 10.1242/jcs.075119

[37] Pellegrino MW, Nargund AM. Signaling the mitochondrial unfolded protein response. Biochim Biophys Acta. 2013;1833(2):410–416. 22445420 10.1016/j.bbamcr.2012.02.019 PMC3393825

[38] Moisoi N, Klupsch K, Fedele V, et al. Mitochondrial dysfunction triggered by loss of HtrA2 results in the activation of a brain-specific transcriptional stress response. Cell Death Differ. 2008;16(3):449–464. 19023330 10.1038/cdd.2008.166

