# Supplementary material for: Mitochondrial serine protease Omi/HtrA2 accentuates brain ischemia/reperfusion injury in rats and oxidative stress injury in vitro by modulating mitochondrial stress proteins CHOP and ClpP and physically interacting with mitochondrial fusion protein OPA1
Source: Bioengineered. 2020 Oct 4;11(1):1058–70. doi: 10.1080/21655979.2020.1822105 (PMC8291814; doi:10.1080/21655979.2020.1822105)
Supplement: Supplemental Material [file KBIE_A_1822105_SM4540.zip › Supplementary figure legends.docx]

**Supplementary figure legends**

**Supplementary Figure 1** The effect of H_2_O_2_ at different concentrations on the viabilities of PC12 cells. Cell viability was determined by MTT assays. Data are presented as mean ± SD (n=3).
